# The Association of a Panel of Biomarkers with the Presence and Severity of Carcinoid Heart Disease: A Cross-Sectional Study

**DOI:** 10.1371/journal.pone.0073679

**Published:** 2013-09-12

**Authors:** Rebecca Dobson, Malcolm I. Burgess, Melissa Banks, D. Mark Pritchard, Jiten Vora, Juan W. Valle, Christopher Wong, Carrie Chadwick, Keith George, Brian Keevil, Joanne Adaway, Joy E. S. Ardill, Alan Anthoney, Uschi Hofmann, Graeme J. Poston, Daniel J. Cuthbertson

**Affiliations:** 1 Neuroendocrine Tumour Group, University Hospital Aintree, Liverpool, Merseyside, United Kingdom; 2 Department of Cardiology, University Hospital Aintree, Liverpool, Merseyside, United Kingdom; 3 Department of Gastroenterology, Institute of Translational Medicine, University of Liverpool, Liverpool, Merseyside, United Kingdom; 4 Neuroendocrine Tumour Group, Royal Liverpool University Hospital, Liverpool, Merseyside, United Kingdom; 5 Manchester Academic Health Sciences Centre, Department of Medical Oncology, The Christie NHS Foundation Trust, Withington, Manchester, United Kingdom; 6 Research Institute for Sport & Exercise Sciences, Liverpool John Moores University, Liverpool, Merseyside, United Kingdom; 7 Department of Clinical Chemistry, University Hospital of South Manchester, Wythenshawe, Manchester, United Kingdom; 8 Regional Peptide Laboratory, Kelvin Building, Royal Victoria Hospital, Belfast, United Kingdom; 9 Medical Oncology, CRUK Clinical Cancer Centre, St James University Hospital, Leeds, Yorkshire, United Kingdom; 10 Medical Oncology, Huddersfield Royal Infirmary, Lindley, Huddersfield, Yorkshire, United Kingdom; 11 Department of Obesity and Endocrinology, Institute of Ageing and Chronic Disease, University of Liverpool, Liverpool Merseyside, United Kingdom; Katholieke Universiteit Leuven, Belgium

## Abstract

**Purpose:**

Metastatic neuroendocrine tumors secrete serotonin and other vasoactive substances that are responsible for carcinoid syndrome and carcinoid heart disease. We sought to evaluate the discriminatory utility of diagnostic biomarkers in determining the presence and severity of carcinoid heart disease in patients with metastatic neuroendocrine tumors.

**Patients and methods:**

A cross-sectional study of patients with neuroendocrine tumors with documented liver metastases and/or carcinoid syndrome between April 2009–October 2012 in 5 tertiary referral centers. Serum was analyzed for Chromogranin A, Chromogranin B and N-terminal pro Brain Natriuretic Peptide (NT-proBNP). Plasma was analyzed for Neurokinin A and 5-Hydroxyindoleacetic acid (5HIAA). Echocardiography was used to determine the presence and severity of carcinoid heart disease. Non-parametric receiver operating characteristic curves were constructed for biomarkers, and the area under the curve determined. The severity of cardiac involvement was correlated with the concentration of each biomarker.

**Results:**

A total of 187 patients were identified of whom 37 (20%) had carcinoid heart disease. Significantly higher median values of all biomarkers were found in the patients with cardiac involvement. NT-proBNP and plasma 5HIAA had the highest areas under the curve for the prediction of carcinoid heart disease [NT-proBNP 0.82 (95% confidence interval 0.74–0.90, p<0.0001) and 5HIAA 0.85 (95% confidence interval 0.78–0.92, p<0.0001]. NT-proBNP was moderately correlated (r = 0.48, p<0.001) whereas plasma 5HIAA was only weakly correlated (r = 0.34, p<0.001) with the echocardiographic severity score.

**Conclusion:**

NT-proBNP and plasma 5HIAA are both sensitive and specific biomarkers for the presence of carcinoid heart disease whereas only NT-proBNP is moderately correlated with disease severity.

## Introduction

Metastatic mid-gut neuroendocrine tumors (NETs) secrete serotonin and a variety of other vasoactive substances that are responsible not only for the characteristic carcinoid syndrome, but also for the significant long-term complication of carcinoid heart disease.

The pathophysiology of carcinoid heart disease has been elucidated from human and animal studies with increased plasma concentrations of serotonin strongly implicated from several lines of evidence. The specific serotonin re-uptake inhibitors fenfluramine and dexfenfluramine, used as appetite-suppressant drugs, and the ergot alkaloids, ergotamine and methysergide, used in the treatment of migraine, are known to cause valvular fibrosis [1], [2]. Secondly, in an *in vivo* rodent model of carcinoid syndrome, in which there are significant increases in plasma serotonin, mice exhibited fibrotic cardiac valvular disease, which was abrogated by somatostatin analogues [3]. Other mechanisms are also thought to contribute to the pathophysiology of the disease, with activin A [4] and connective tissue growth factor [5] both associated with the development of carcinoid heart disease. Carcinoid heart disease is characterized by thickening of the tricuspid and pulmonary valves, resulting in regurgitation and/or stenosis of the affected valve [1]. Any or all of the cardiac valves can be affected, with tricuspid regurgitation being the most frequently observed pathology. Involvement of the left sided valves is mainly seen in patients with patent foramen ovale or bronchial carcinoid [6].

Detecting the presence of carcinoid heart disease is important in determining the most appropriate management strategy, particularly with regard to surgical resection of the primary or metastatic tumor [7]. Carcinoid heart disease also has prognostic significance for long term survival [8]. Screening for cardiac involvement is an integral part of the initial assessment and subsequent surveillance protocol in patients with metastatic NETs. Current European Neuroendocrine Tumor (ENETS) guidelines [9] advocate transthoracic echocardiography for all patients with carcinoid syndrome, although the recommended screening interval is not stated. The identification of a sensitive and specific biochemical marker that can predict the presence and severity of carcinoid heart disease may direct more rational use of echocardiographic screening.

Previous studies have identified a variety of biomarkers for carcinoid heart disease. The most useful to date has been N-terminal pro-brain natriuretic peptide (NT-proBNP) which has both diagnostic and prognostic significance for cardiac involvement [10], [11]. Bhattacharyya *et al* [12] and subsequently Korse *et al* [10], found similar cut-off levels of 260 pg/ml and 280 pg/ml for the detection of carcinoid heart disease in patients with carcinoid syndrome. The concentration of Chromogranin A (CgA), present in the chromograffin granules of neuroendocrine cells, has also been significantly associated with the prevalence of and survival from carcinoid heart disease [10]. Furthermore, Neurokinin A (NkA), a tachykinin that is stored and secreted by midgut NETs, is a strong and independent predictor of premature death in these patients [13]. As progression of carcinoid heart disease is thought to be related to serotonin, it might be expected that plasma serotonin, or one of its metabolites, is a useful biochemical correlate of disease severity. Urinary levels of 5-Hydroxyindoleacetic acid (5HIAA), the main metabolite of serotonin, have been linked with the presence and progression of carcinoid heart disease [14], [15] but there is no literature on the utility of plasma 5HIAA, a more practical measure. Furthermore we have found no studies of patients with metastatic NETS that compare the discriminatory ability of a panel of biomarkers in the assessment of the presence and severity of carcinoid heart disease.

The aim of the study was to determine which biomarker was best associated with the presence and severity of carcinoid heart disease in patients with metastatic NETs.

## Materials and Methods

### Participants

All patients with non-pancreatic NETs who visited the outpatient department of one of five tertiary referral NET centers (University Hospital Aintree NHS Trust, Liverpool, Royal Liverpool and Broadgreen University Hospitals NHS Trust, Liverpool, The Christie Hospital NHS Foundation Trust, Manchester, St James’s University Hospital, Leeds and Huddersfield Royal Infirmary NHS Foundation Trust, Yorkshire) during the study period of April 2009 - October 2012 were eligible.

The inclusion criteria were diagnosis of a NET, with liver metastases and/or carcinoid syndrome (episodes of cutaneous flushing, diarrhea or wheezing) by a specialist neuroendocrine team and clear visualization of all four valves at echocardiography to enable comprehensive assessment of valve leaflet morphology, mobility and thickening. The exclusion criteria were prosthetic heart valves or inadequate visualization of valves/poor echocardiographic windows such that an echocardiographic score could not be determined. Liverpool Research Ethics Committee approved the study and all patients gave written informed consent.

Diagnosis of a NET was based on histological evidence where available, with characteristic immunostaining for CgA and synaptophysin. Histological grading was based on the Ki67 proliferative index. Where histological evidence was not available the diagnosis was made on the basis of characteristic appearances on cross-sectional imaging (computed tomography and/or magnetic resonance) and functional (^111^In-Octreotide scan) imaging.

All patient records were reviewed and the following extracted: histological diagnosis, tumor proliferative index, duration of disease, primary tumor site, presence or absence and size of hepatic metastases, presence of carcinoid syndrome, use of proton pump inhibitor therapy (a known confounder of CgA levels) and therapeutic interventions. In addition a questionnaire was used to identify New York Heart Association (NYHA) category.

### Blood Samples and Assays

Venous (non-fasting) blood samples were taken from each patient at the time of echocardiography. Plasma and serum were separated by centrifugation (3,500 rpm) and stored at −80°C until further analysis.

Serum CgA, upper limit of normal (ULN) 3 ng/ml, (synthetic porcine pancreastatin, Peninsula, CA, USA, comparable to the Cis-Bio and DAKO assays) [16] and Chromogranin B (CgB), ULN 12 ng/ml, (GAWK immunoassay, Cambridge Research Biochemical, Cambridge, UK with an intra-assay variation of 12.3% at 12.4 pmol/l to 6.8% at 134 pmol/l) and a corresponding inter-assay variation of 22 and 11% respectively [17] were measured in 157 patients. Serum NT-proBNP, ULN 146 ng/L [18], (electrochemiluminescence technology on the fully automatic Elecsys**®** analyzer) with intra-assay precision below 4% and an inter-assay precision below 5% at concentrations above 70 pg/ml (Roche Diagnostics) was measured in 172 patients.

Plasma 5HIAA, ULN 23 ng/ml, LC-MS/MS method (comparable to that used by Tellez et al [19]) with QuanLynx™ software (Waters, Watford, UK) with an inter-assay coefficient of variation of 2.6–9.8% and a intra-assay variation of 2–4.7%) [20] was measured in 160 patients. Plasma NkA, ULN 20 ng/L, (an in-house radioimmunoassay developed by the Regional Regulatory Peptide Laboratory, Royal Victoria Hospital, Belfast with an inter-assay coefficient of variation of 6.4–8.4% and an intra-assay coefficient of variation of 1.6–4.6% [21] was measured in 143 patients. All biochemical measurements were made without knowledge of the clinical status of the patient. Due to patient-specific and analytical factors not all patients had every biomarker concentration determined.

### Trans-thoracic Echocardiography Image Acquisition

All echocardiograms were performed and analyzed by one of two experienced operators, with the patient positioned in the left lateral position on a reclining couch using a GE Vivid 7 or Vivid Q machine (2.5 MHz phased array transducer, Horten, Norway). Valve anatomy and function were assessed in the parasternal long and short axes, and apical 4 chamber, 2 chamber and long axes. Evaluation included 2 dimensional, M-mode, pulsed and continuous wave Doppler and pulsed-wave tissue Doppler imaging. Video loops were acquired triggered to the ECG (3 cardiac cycles) and saved digitally for subsequent off line analysis (Echopac V9.01, GE, Horten, Norway).

### Trans-thoracic Echocardiography Image Analysis and Interpretation

A diagnosis of carcinoid heart disease was decided by the operator based on consensus guidelines [9]. Valve regurgitation and stenosis was quantified according to the American College of Cardiology Guidelines [22]. Tricuspid stenosis was quantified according to the mean gradient across the valve (mild 1–5 mmHg, moderate 5–8 mmHg, severe >8 mmHg). Pulmonary stenosis was quantified according to the maximum gradient across the valve (mild <25 mmHg, moderate 25–50 mmHg and severe >50 mmHg). Right ventricular size and function was assessed according to the American Society of Echocardiography guidelines [23]. This enabled calculation of a previously validated echocardiographic score [12] incorporating assessment of all four cardiac valves. Leaflet thickening, mobility and morphology, valvular regurgitation and stenosis, and right ventricular size and function were graded, with higher scores indicating more severe valvular pathology.

**Figure 1:**
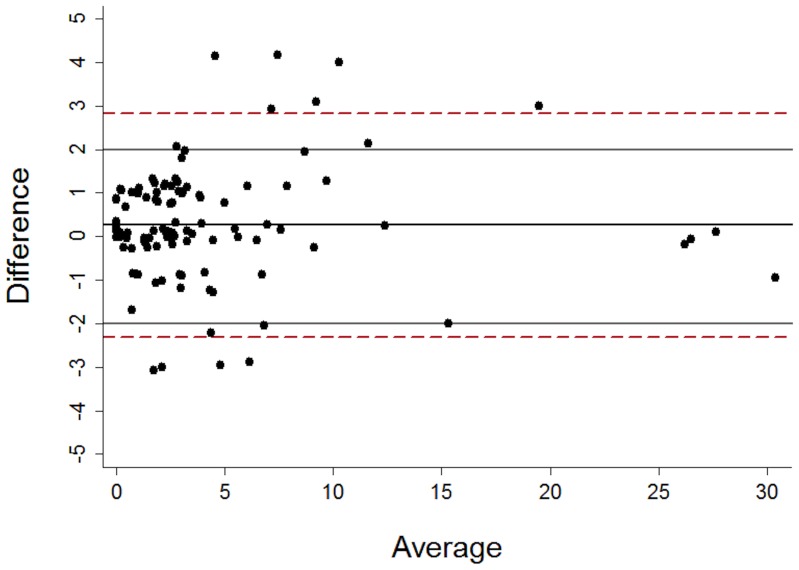
Bland Altman plot demonstrating degree of inter-observer agreement in assigning echocardiographic scores.

To ensure inter-observer agreement a randomly selected sub-group of 108 echocardiographic studies (57%) were cross-checked by a different observer blinded to the initial echocardiographic score. With a maximum score of 66, scores within 2 points were considered to be concordant. In 98 studies the assigned scores were concordant, in ten there was a discrepancy of >2 points, (mean difference 0.269, see figure 1). Where there was score discordance, a third independent observer analyzed the echocardiographic study, blinded to the other scores. An average of the two most concordant scores was then used for these ten echocardiographic studies.

### Recruitment

A target of 200 patients was decided at the outset of the study based upon a conservative estimate of 20% incidence of carcinoid heart disease in our patient population. Two hundred patients would be sufficient to demonstrate that an area under the receiver operating characteristic (ROC) curve of 0.65 was better than chance (0.5) with 80% power and at the 5% significance level (with no correction for multiple comparisons). Target recruitment was also based upon the cumulative total of patients attending each of the tertiary referral centers and the availability of a dedicated Cardiology Research Registrar to recruit and study the patients.

### Statistical Analysis

Comparisons between continuous variables in the carcinoid heart disease and the non-carcinoid heart disease group were made using the Mann-Whitney U test, as our data did not satisfy the assumption of equal variances for the two-sample t-test. Categorical variables were compared using Chi-square test, or Fisher’s exact test where cell counts were insufficient. Since relationships between the concentration of each biomarker and echocardiographic score were clearly non-linear, Spearman’s rank correlation coefficient (r) was used. For graphical display of bivariate correlations the natural logarithms of the score and biomarker concentrations were used to aid clarity. Non-parametric ROC curves were constructed to assess the ability of each biomarker to discriminate between those patients with and without a diagnosis of carcinoid heart disease. Multivariable logistic regression was used to assess whether any combination of assays was able to better discriminate between patients with and without carcinoid heart disease compared with any single assay. The number of candidate variables considered was limited to 4 to satisfy the 10 events per variable rule of thumb (Van Belle, 2002). The final model was obtained using a stepwise model selection procedure based on Akaike’s Information Criterion (AIC). Statistical analyses were performed using Stata/IC 12.0 software (StataCorp LP, College Station, TX, USA). A p value of <0.05 was considered statistically significant. No correction was made for multiple comparisons.

## Results

### Patients Recruited

Two hundred and eighteen patients were invited to participate. Thirty-one patients were excluded due to a variety of reasons: Inadequate visualisation of all four valves preventing calculation of echocardiographic score (26 patients), presence of prosthetic valves (3 patients), inability to provide informed consent (1 patient), and 1 patient declined the invitation to participate, resulting in a total of 187 patients studied. The baseline characteristics of the patients are shown in Table 1. One hundred and sixty-six patients had a histologically confirmed NET. Of the 121 patients with a Ki67 proliferative index reported: 69 patients had a grade 1 tumor (Ki67<2%), 50 patients had grade 2 tumors (Ki67 2–20%) and 2 patients’ tumors were grade 3 (Ki67>20%). Thirty-one patients were newly diagnosed (<3 months since presentation) with a metastatic NET while 156 had an established diagnosis with a median duration of 56 months (interquartile range 35–93 months).

**Table 1:** Baseline characteristics of all patients, and carcinoid heart disease status.

Variable	All patients (n = 187)	No carcinoid heart disease (n = 150)	Carcinoid heart disease (n = 37)
***Demographics***			
Age (years)†	67±10	66±10	68±12
Male sex	101 (54%)	84 (56%)	17 (44%)
Body mass index (kg/m^2^)†	25.9±5.0	26.6±4.5*	22.1±6.0*
***Clinical Characteristics***			
Histologically confirmed diagnosis	166 (89%)	136 (90%)	30 (83%)
Duration of disease (months)◊	42 (26–74)	47 (28–79)	29 (9–63)
New NET diagnosis at time of echocardiogram	32 (17%)	23 (15%)	9 (24%)
Proton pump inhibitor therapy	54 (29%)	46 (30%)	8 (22%)
Liver metastases	156 (83%)	119 (79%)*	37 (97%)*
Carcinoid syndrome	120 (64%)	95 (63%)	25 (68%)
***Site of primary tumor***			
Small bowel	132 (71%)	108 (72%)	24 (67%)
Large bowel	12 (6%)	11 (7%)	1 (3%)
Lung	5 (3%)	5 (3%)	0
Other	7 (4%)	4 (3%)	3 (8%)
Unknown	31 (16%)	23 (15%)	8 (22%)
***NYHA Class***			
I	133 (71%)	117 (77.5%)*	16 (44%)*
II	49 (26%)	33 (22%)	16 (44%)
III	4 (2%)	1 (0.5%)	3 (8%)
IV	1 (1%)	0	1 (3%)
***Therapeutic Intervention***			
SSA therapy	89 (48%)	68 (45%)	21 (58%)
Small bowel resection	118 (63%)	101 (67%)*	17 (47%)*
Hepatic resection	19 (10%)	18 (12%)	1 (3%)

† Mean ± standard deviation.

◊ Median and interquartile range.

* p<0.05.

NYHA New York Heart Association.

SSA Somatostatin Analogue.

There were no significant differences with respect to age, gender, or primary tumor site between the groups with and without carcinoid heart disease. Furthermore there was no difference in known duration of disease between the two groups (median duration 47 v 29 months, p = 0.102). The proportions of patients with liver metastases and symptoms of heart failure were higher in those with carcinoid heart disease than in those without. Those patients who had undergone previous primary tumor resection had a significantly lower prevalence of carcinoid heart disease than those without previous primary tumour resection, although this was not the case for patients who had or had not undergone hepatic resection.

### Echocardiography Results

Thirty-seven patients (20%) had echocardiographic evidence of carcinoid heart disease. The median echocardiographic score in those with cardiac involvement was 12 (interquartile range [IR] 8–21), compared to a median of 2 (IR 1–3) in those without cardiac involvement. There were 24 cases of single valve involvement (tricuspid in 23 cases, pulmonary in 1), 12 cases of dual valve pathology, 1 patient with three valves involved and no cases with all four cardiac valves affected. The right heart was dilated in 34 cases and right ventricular function was affected in 10 cases. Eight patients (4%) had evidence of left sided carcinoid heart disease, all of which had patent foramen ovale. The echocardiographic score was weakly correlated with NYHA class, i.e. patient symptoms (Spearman r = 0.22; p 0.002).

### Predictive Value of Biomarkers for Diagnosis of Carcinoid Heart Disease

**Figure 2:**
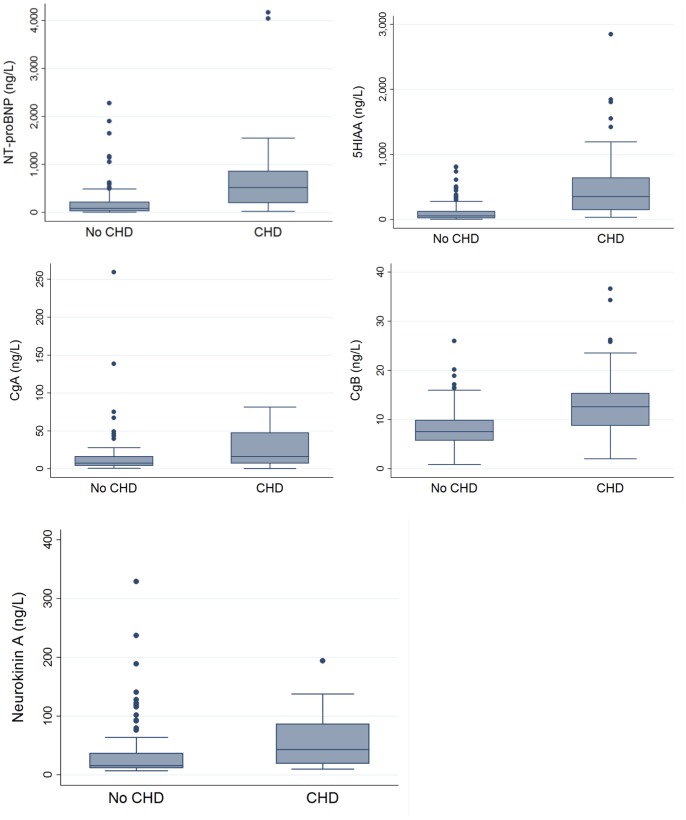
Box plots demonstrating spread of results for all 5 biomarkers.

**Figure 3:**
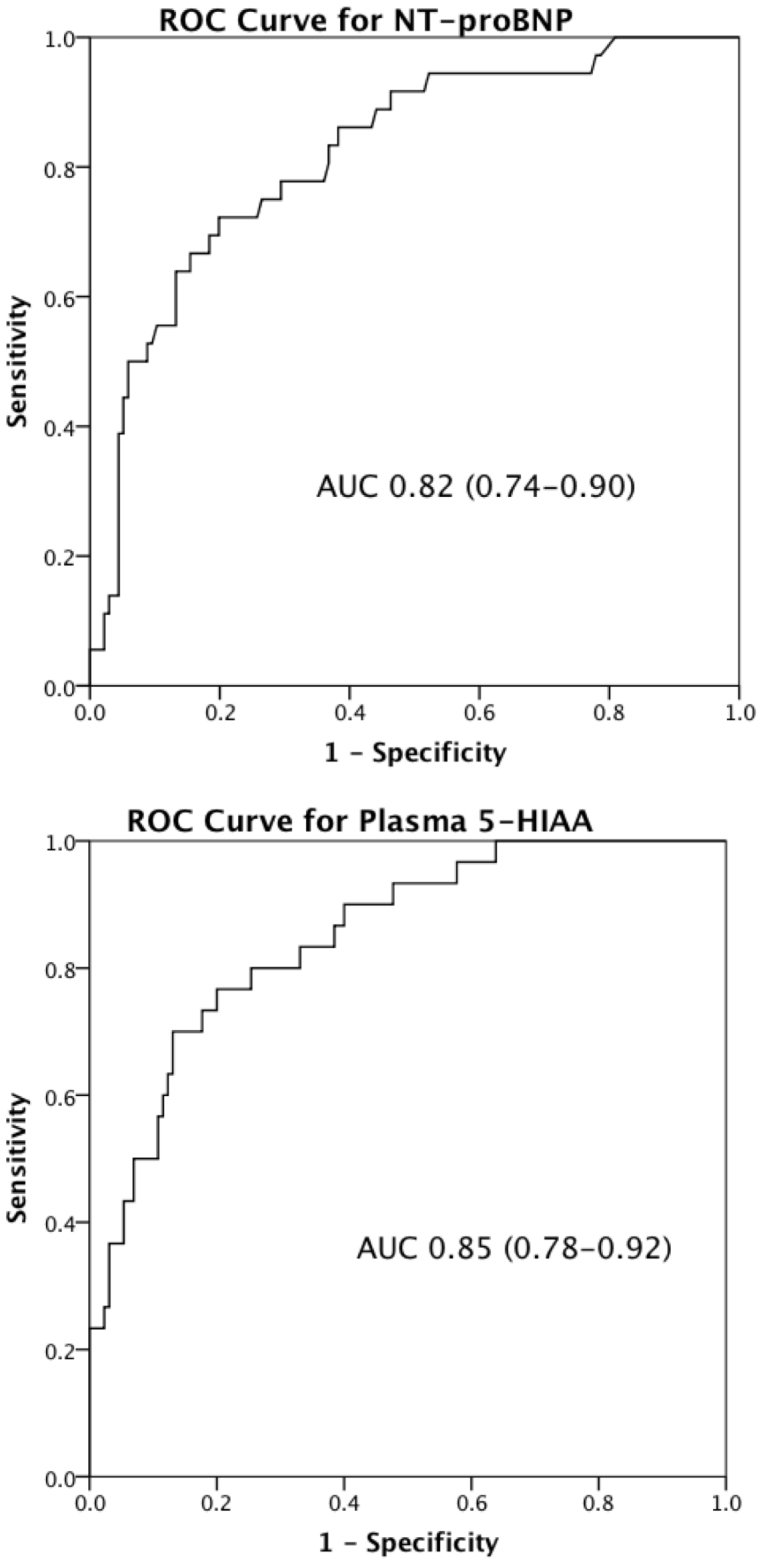
Receiver operator curves for plasma 5HIAA and NT-proBNP with area under curves (AUC) and 95% confidence intervals presented.

Significantly higher median values of all 5 biomarkers were found in the patients with carcinoid heart disease compared to those without, (NT-proBNP 474 vs. 79 ng/L, 5HIAA 353 vs. 57 ng/ml, CgA 16 vs. 7 ng/ml, CgB 13 vs. 8 ng/ml and NkA 27 vs. 16 ng/L, Figure 2). Plasma 5HIAA and NT-proBNP had the greatest areas under the curve (AUC) (0.85 plasma 5HIAA and 0.82 NT-proBNP, Figure 3). The AUCs for CgA, CgB and NkA were 0.69 (0.57–0.80), 0.77 (0.67–0.88) and 0.72 (0.62–0.82) respectively.

The effect of age, sex and creatinine on NT-proBNP was investigated using a multivariable prediction model. NT-proBNP remained statistically significant whereas age, gender and creatinine did not (p = 0.599, 0.521 and 0.749 respectively). A stepwise model selection method (minimizing Akaike’s Information Criterion) demonstrated that age, gender and creatinine do not offer any additional information to discriminate between patients with and without carcinoid heart disease (univariate p-values of 0.277, 0.300 and 0.822 respectively).

The cut-off points for NT-proBNP and plasma 5HIAA with their respective sensitivities and specificities in our patient cohort are illustrated in table 2. The improvement in discriminatory power by inclusion of additional assays into a final logistic regression model was negligible (less than 5-point change in AIC; direct comparison of full model with plasma 5HIAA yielded p = 0.68). For every 50 ng/l increase in NT-proBNP concentration, the odds of carcinoid heart disease increases by 11% (95% confidence interval 5–17%, p<0.0005) and for every 50 ng/ml increase in plasma 5HIAA the odds of carcinoid heart disease increases by 26% (95 confidence interval 14–39%, p<0.0005).

**Table 2:** Diagnostic accuracy of NT-proBNP & plasma 5HIAA.

NT-proBNP cut point(ng/L)	Sensitivity	Specificity	Correctly classified	Proportion abovecut-point		LR+	LR−
100	88	54	55%	55%		1.9	0.2
200	74	73	73%	37%		2.8	0.3
300	67	82	79%	28%		3.6	0.4
400	61	87	81%	23%		4.6	0.4
500	50	93	84%	16%		6.8	0.5
600	42	95	84%	13%		8.1	0.6
**5HIAA cut point (ng/ml)**							
50	93	45	54%		96%	1.7	0.1
100	80	68	71%		82%	2.5	0.3
150	73	80	79%		74%	3.7	0.3
200	70	85	83%		68%	4.8	0.4
250	67	87	83%		63%	5.1	0.4
300	57	88	83%		58%	4.9	0.5
350	50	92	84%		54%	5.9	0.5

LR+ Positive likelihood ratio.

LR− Negative likelihood ratio.

### Correlation between Biomarkers and Echocardiographic Severity Score

**Figure 4:**
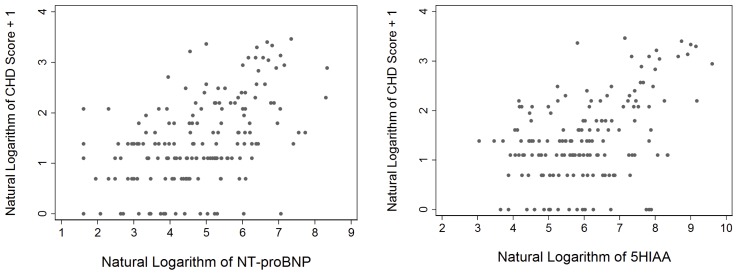
Scatter diagram demonstrating correlations of NT-proBNP and plasma 5HIAA with the echocardiographic score.

There were significant correlations between the echocardiographic severity score and concentrations of all biomarkers except CgA. The score correlated most significantly with NT-proBNP (Spearman r = 0.48; p<0.0001) while for the other biomarkers the correlation coefficients were low: plasma 5HIAA (Spearman r = 0.34; p<0.0001), CgA 0.09 (p = 0.25), CgB 0.29 (p<0.0001) and NkA 0.19 (p = 0.03) (Figure 4).

## Discussion

Our study is the first large prospective clinical study of patients with metastatic NETs to compare the discriminatory ability of a panel of biomarkers in the assessment of the presence and severity of carcinoid heart disease. We have demonstrated that the median concentrations of all five biomarkers are significantly higher in patients with carcinoid heart disease; however NT-proBNP and plasma 5HIAA had the greatest discriminatory value in the diagnosis of carcinoid heart disease, with NT-proBNP most closely correlating with its severity. Thus in this panel of biomarkers for use in patients with metastatic NETs, NT-proBNP concentration is the most useful biochemical assay for clinicians regarding the presence and severity of cardiac involvement. This finding is consistent with the most recent ENETS and North American Neuroendocrine Tumor (NANETs) consensus guidelines advocating the use of natriuretic peptides where available [24], [25].

The optimum cut-off point for NT-proBNP is determined by the relative importance of the sensitivity and specificity of the test. In this circumstance, sensitivity assumes greater importance than specificity, as it is preferable to perform echocardiography on too many patients, rather than miss a diagnosis of carcinoid heart disease. Unlike the results of Korse *et al*, we found no incremental benefit in combining NT-proBNP with CgA, although there was minor incremental benefit in combining NT-proBNP and plasma 5HIAA in terms of increasing the diagnostic accuracy of the test. A single variable model was considered to be favorable due to its enhanced ease of use in clinical practice. In our series, the cut-off point for NTproBNP of 260 pg/ml suggested by Bhattacharya, would have a sensitivity of 69% and a specificity of 80%, meaning that 11 patients with carcinoid heart disease would have escaped detection.

NT-proBNP is released from cardiac myocytes with myocyte stretch being the main stimulus for its synthesis and secretion [18]. Elevated levels reflect increased wall tension and pressure, making its measurement of value in carcinoid heart disease. Our finding of the correlation between the degree of elevation of plasma 5HIAA and the development of carcinoid heart disease gives mechanistic insight into the likely contribution of serotonin to the pathogenesis of cardiac involvement. The use of somatostatin analogues (SSAs) that are known to reduce circulating levels of serotonin may be expected to be protective against the development of carcinoid heart disease and as yet, we were unable to confirm this hypothesis. This negative association likely reflects a treatment bias, in that patients with a greater tumor burden are more likely to require treatment with SSAs. Furthermore 83% of our cohort had liver metastases and 64% had carcinoid syndrome but only 48% received SSAs which may have influenced our findings. Prospective human studies are needed to determine whether SSAs can prevent or decrease severity of carcinoid heart disease, although the anti-proliferative effect of SSAs may result in an increasingly higher proportion of patients with metastatic NETs receiving them.

We found a poor correlation between CgA and the severity of carcinoid heart disease which may be because CgA is falsely elevated by proton pump inhibitor (PPI) therapy in healthy individuals [26], and those with NETs [27]. Thirty percent of patients who did not have carcinoid heart disease were taking PPI therapy, which undoubtedly will have influenced the CgA results. CgB is known to regulate cardiomyocyte signalling pathways that mediate hypertrophy and heart failure [28]. Furthermore CgB is not affected by renal function or PPI therapy [29]. These factors may explain why we found CgB to be more sensitive and specific than CgA for carcinoid heart disease. NkA is a specific marker for tumours of the mid-gut [13] and as we included a minority of patients with NETs not of mid-gut origin, this may have affected the NkA results for our patient cohort.

The distribution of NYHA categories in patients with carcinoid heart disease (88% of patients in categories 1 and 2) demonstrates that the disease is often clinically silent, with no discernable attributable symptoms. This is reflected in the weak correlation of the echocardiographic score with NYHA class. We advocate screening all patients with metastatic NETs to facilitate timely diagnosis and management of carcinoid heart disease, and also to assist with the optimum timing of other therapeutic interventions for NETs, e.g. hepatic resection of metastases 7.

While the strength of our study is the large population of NET patients, carefully characterized prospectively from a multi-modality perspective with a comparison of multiple biomarkers, we acknowledge some limitations. Most significantly, our exclusion of patients in whom it was not possible to visualise all 4 cardiac valves may have led to an underestimate in the prevalence of carcinoid heart disease. Additionally, we currently lack data on the utility or discriminatory ability of these biomarkers in determining the mortality from carcinoid heart disease. Further large, long term prospective studies are required to provide robust evidence of the prognostic role of NT-proBNP in the associated mortality from carcinoid heart disease.

Our findings suggest that NT-proBNP or plasma 5HIAA could direct the use of transthoracic echocardiography in the assessment of patients with metastatic NETs. We propose that all such patients undergo a baseline echocardiogram at diagnosis or initial assessment, and if there is no evidence of cardiac involvement, echocardiography is only repeated in the event of an increase in NT-proBNP. Such an approach would necessitate a proportion of patients without carcinoid heart disease being screened unnecessarily but would successfully identify the vast majority of patients with the disease. We recognise that NT-proBNP is not highly specific and therefore unsuitable for monitoring patients with an elevated NT-proBNP attributable to a different pathology. In this scenario, it is necessary to resort to annual echocardiography.

In summary, we have demonstrated that NT-proBNP and plasma 5HIAA are equally useful in determining the diagnosis of carcinoid heart disease. NT-proBNP correlates better with severity of disease and may be superior to determine progression but both biomarkers could be used as part of the routine surveillance protocol in patients with metastatic NETs.
